# Comprehensive Biological and Chemical Evaluation of Two *Seseli* Species (*S. gummiferum* and *S. transcaucasicum*)

**DOI:** 10.3390/antiox10101510

**Published:** 2021-09-24

**Authors:** Gokhan Zengin, Dejan Stojković, Mohamad Fawzi Mahomoodally, Bibi Sharmeen Jugreet, Mehmet Yavuz Paksoy, Marija Ivanov, Uroš Gašić, Monica Gallo, Domenico Montesano

**Affiliations:** 1 Department of Biology, Science Faculty, Selcuk University, 42130 Konya, Turkey; 2 Department of Plant Physiology, Institute for Biological Research “Siniša Stanković”—National Institute of Republic of Serbia, University of Belgrade, Bulevar Despota Stefana 142, 11000 Belgrade, Serbia; marija.smiljkovic@ibiss.bg.ac.rs (M.I.); uros.gasic@ibiss.bg.ac.rs (U.G.); 3 Department of Health Sciences, Faculty of Medicine and Health Sciences, University of Mauritius, Réduit 80837, Mauritius; f.mahomoodally@uom.ac.mu (M.F.M.); sharmeenjugs@gmail.com (B.S.J.); 4 Department of Medical Services and Techniques, Medical Documentation and Secretaryship Programme, Tunceli Vocational School, Munzur University, 62000 Tunceli, Turkey; mypaksoy@gmail.com; 5 Department of Molecular Medicine and Medical Biotechnology, University of Naples Federico II, Via Pansini 5, 80131 Naples, Italy; mongallo@unina.it; 6 Department of Pharmacy, University of Naples Federico II, Via D. Montesano 49, 80131 Naples, Italy

**Keywords:** *Seseli*, polyphenolic, antioxidant, enzyme inhibitors, antibacterial, wound healing

## Abstract

*Seseli* L. is one of the largest genera of the Apiaceae family widely known for their traditional uses as herbal remedies. In the present study, the methanolic and water extracts of two *Seseli* species, *S. gummiferum* and *S. transcaucasicum* were evaluated for their bioactive contents and biological activities. The total phenolic and flavonoid contents in the extracts ranged from 19.09 to 24.33 mg GAE/g and from 0.45 to10.09 mg RE/g, respectively. Moreover, while narcissin was detected as the most abundant component in the methanolic extract of *S. transcaucasicum* (261.40 µg/g), chlorogenic acid was identified as the major component in all the other extracts, although a high amount was also present in the methanolic *S. transcaucasicum* extract (107.48–243.12 µg/g). The total antioxidant capacity was also determined by the phosphomolybdenum assay (0.66–1.18 mM TE/g). Other antioxidant assays such as the radical scavenging assays (DPPH: 5.51–11.45 mg TE/g; ABTS: 43.46–51.91 mg TE/g), reducing assays (CUPRAC: 41.67–53.20 mg TE/g; FRAP: 31.26–34.14 mg TE/g), as well as the metal chelating activity assay (14.38–38.57 mg EDTAE/g) were conducted. All the extracts showed inhibitory potential against the enzyme’s amylase (0.12–0.78 mM ACAE/g), acetyl- and butyryl-cholinesterase (0.15–9.71 mg GALAE/g), while only the methanolic extracts acted as inhibitors of tyrosinase (107.15 and 109.37 mg KAE/g) and only the water extract of *S. gummiferum* displayed anti-glucosidase activity (0.13 mM ACAE/g). Interestingly, the methanolic extracts of both *Seseli* species showed lower cytotoxicity towards HaCaT cells (IC_50_: >500 µg/mL), compared to the water extracts (IC_50_: 267.8 and 321.41 µg/mL). Besides, only the methanolic extracts showed a slight wound healing effect (28.21 and 31.23%). All extracts showed antibacterial action against *Staphylococcus lugdunensis* (minimum inhibitory and bactericidal concentrations: 0.025–2 mg/mL). *S. gummiferum* methanolic extract, which exhibited the highest antibacterial potency was found to inhibit adhesion and invasion of *S. lugdunensis* to HaCaT cells as well. Taken together, this study demonstrated the two *Seseli* species to harbour interesting bioactive components, in particular polyphenolics and to exhibit several biological properties that could be further investigated for their potential exploitation as healing agents as supported by various traditional medicinal uses.

## 1. Introduction

*Seseli* L. is an important genus comprising of many aromatic species (about 140 taxa) used in traditional medicine due to its recognized therapeutic properties. Several *Seseli* species are used in folk medicine as herbal drugs for several purposes including to treat common cold, inflammation, pain as well as an antiflatulent [1]. Moreover, species of this genus have been reported to possess anthelmintic, carminative, stomachic and stimulant properties [2]. Some representatives of *Seseli* genus have also been used in the treatment of central nervous system disorders such as epilepsy [3]. Furthermore, some members of the genus *Seseli* have been widely employed in traditional medicine owing to their antibacterial, antifungal, and insect repellent activities [4], while others are known as being antispasmodic and diuretic [5].

In light of the ethnobotanical uses; several studies have been conducted on the biological activities of the members of this genus. Previously, antibacterial, anticancer, anti-inflammatory and antinociceptive effects have been confirmed for several *Seseli* species [2,6,7,8]. Furthermore, a number of scientific reports have demonstrated *Seseli* species to contain volatile oils with important pharmacological potentials. For instance, various studies have shown essential oils from *Seseli* species to possess antimicrobial as well as antioxidant properties [9,10,11]. In another study, the safety of the essential oil from a *Seseli* species (*S. tortuosum*) was reported for doses with anti-inflammatory activity [1].

Besides, the genus *Seseli* is known to be rich in coumarins, a number of which are recognized for their medicinal properties [12,13,14,15]. Attempts were also made to explore the detailed underlying mechanism of coumarins isolated from *Seseli* species, as active pharmacological agents. Notably, the anti-inflammatory properties of samidin from *S. resinosum* through suppression of NF-jB and AP-1-mediated-genes in LPS-stimulated RAW 264.7 cells, were reported [16]. Additionally, another two new coumarins from *S. resinosum*, were found to exhibit anti-inflammatory effects through the inhibition of pro-inflammatory enzymes and cytokines [17]. Moreover, previously the lignans seselinone and eudesmin, isolated from the aerial parts of *S. annuum*, displayed cytotoxic activity against C6 rat glioma cell cultures [18].

Hence, the scientific data collected so far on several *Seseli* species point out the tremendous healing potentials of these plants that can benefit human health and be used in the management of various diseases, as supported by their widespread uses in traditional medicine. To the best of our knowledge, however, the chemical composition, and biological activities of most members of the *Seseli genus* have not yet been investigated. Thus, in this study, the bioactive contents and polyphenolic profile of methanolic and water extracts of two such uninvestigated species of *Seseli* (*S. gummiferum* and *S. transcaucasicum*) were determined and their various biological potentials (antioxidant, enzyme inhibition, cytotoxic, wound healing and antibacterial properties) were evaluated.

## 2. Materials and Methods

### 2.1. Plant Materials and Extraction Procedure

*Seseli* species were collected in July 2020 from Turkey (*S. gummiferum* Pall. ex Sm.: Akseki, Antalya, Gidefi Mountain, 1800 m and *S. transcaucasicum* (Schischk.) Pimenov & Sdobnina: Van, between Güzelsu and Başkale, Güzeldere location, 2700 m). Voucher specimens were deposited in Munzur University (Voucher numbers: Paksoy 1860 and Paksoy 1414, respectively). The plant materials (aerial parts) were carefully dried in a shade place for 10 days and then they were powdered with a laboratory mill.

For each species, two solvents were used in the preparation of extracts. To prepare the methanolic extracts, the plant materials (5 g) were macerated with 100 mL of methanol in a magnetic stirrer for 24 h at room temperature. After that, the mixtures were filtered and then evaporated to remove the solvents. To prepare water extracts, powdered plant materials (5 g) were infused in boiled water (100 mL) for 15 min. The obtained water extracts were filtered and then lyophilized to remove water. All extracts were stored at 4 °C until further analysis.

### 2.2. Phytochemical Composition

Total phenolic content (TPC) and total flavonoid content (TFC) were determined according to previously described methods [19,20]. TPC was expressed as mg gallic acid equivalents (GAE)/g dry extract, whereas TFC was expressed as mg rutin equivalents (RE)/g dry extract.

### 2.3. UHPLC(-)HESI-QqQ-MS/MS Targeted Metabolic Analysis

Quantification of the targeted compounds was performed using Dionex Ultimate 3000 Ultra-High-Performance Liquid Chromatography (UHPLC) system (Thermo Fisher Scientific, Bremen, Germany) connected to a triple-quadrupole (QqQ) mass spectrometer (MS) (TSQ Quantum Access Max, Thermo Fisher Scientific, Basel, Switzerland).

Syncronis C18 aqua analytical column (100 × 2.1 mm) with 1.7 μm particle size (Thermo Fisher Scientific, Waltham, MA, USA) was used for the chromatographic separation. The flow rate and composition of the mobile phases, as well as the gradient elution program were previously described by Radović, et al. [21]. The mass detector was equipped with heated electrospray ionization (HESI) source operating in the negative ionization mode. The parameters of HESI source and the other mass detector settings were previously described in the study of Božunović, et al. [22]. The compounds of interest were identified and quantified by direct comparison with commercial standards. For each compound, the multiple mass spectrometric scanning modes, including full scanning (FS), and product ion scanning (PIS) were performed. The collision-induced fragmentation experiments were performed using argon as the collision gas, and the collision energy was varied depending on the compound. The time-selected reaction monitoring (tSRM) experiments for quantitative analysis were performed using two MS^2^ fragments for each compound that were previously defined as dominant in the PIS experiments. The total amounts of each compound were evaluated by calculation of the peak areas and are expressed as µg/g.

### 2.4. Antioxidant and Enzyme Inhibitory Assays

By referring to the methods of Uysalet al. [20], the radical scavenging and reducing power abilities of the extracts were evaluated using ABTS, DPPH, CUPRAC and FRAP assays. Likewise, ferrous ion chelating and phosphomolybdenum assays were performed. The results were expressed as equivalents of Trolox (TE) or ethylenediaminetetraacetic acid (EDTA). The ability of the extracts to inhibit the effects of a panel of key enzymes, such as cholinesterases, α-glucosidase, α-amylase and tyrosinase, were investigated according to the protocols described by Grochowski et al. [19]. The enzyme inhibitory activities of the extracts were expressed as equivalents of acarbose for α-glucosidase and α-amylase, galantamine for cholinesterases, and kojic acid for tyrosinase.

### 2.5. Cytotoxicity towards HaCaT Cells

The cytotoxic effect of the extracts was investigated on a spontaneously immortalized skin keratinocyte cell line (HaCaT) by employing crystal violet dye, as reported previously [23], with some modifications. PBS solution was used to dissolve the extracts (8 mg/mL). HaCaT cells were prepared for the assay according to published procedures [24]. The results were expressed as IC_50_ value, indicating 50% of cell viability when compared with untreated control. The solvent was used as a negative control.

### 2.6. Scratch-Wound Healing Assay

The procedures used for the assay were described in detail by Stojković, et al. [25] and the experiment was performed with some modifications. HaCaT cells were grown until confluence was reached. Pipetman tips of 200 μL were used to scratch the cell’s monolayer. Reduced DMEM was used, containing IC_25_ concentration of extracts as determined in the cytotoxicity assay. Cell migration was monitored with Nikon Eclipse TS2 (Amsterdam, The Netherlands) 24 h after the wound was made and treated. Untreated control was used to measure wound closure under these conditions, without the addition of extracts. The results were presented as percentages of wound closure during the exposure to the tested extracts.

### 2.7. Antibacterial Activity

The strain used in the assay was the skin isolate, *Staphylococcus lugdunensis*. The antibacterial activity of different extracts was evaluated by the microdilution method in 96 well microtiter plates. The minimum inhibitory concentrations (MIC) and minimal bactericidal concentrations (MBC) of the bacterial species were determined as described by Kostić, et al. [26]. Streptomycin was used as the positive control.

### 2.8. HaCaT Cell Invasion and Adhesion Assay

For further assays, the methanolic extract of *S. gummiferum* was selected due to its lowest MIC towards *S. lugdunensis* (0.5 mg/mL) compared to other extracts determined in the antibacterial assay. Assessment of the extract ability to reduce adhesion capacity of *S. lugdunensis* towards HaCaT cells was determined as described in the study of Ahmed et al. [24], with some modifications.

For adhesion assay, the confluent monolayer of HaCaT cells was grown in 24-well plates with adhesive bottom. Medium was removed and fresh FBS free DMEM with extract was added and plate was incubated at 37 °C for 15 min. Afterwards 100 µL of *S. lugdunensis* culture (10^8^ CFU/mL) was added to the wells. The mixture was incubated at 37 °C for 1 h. The cells were washed three times with FBS free DMEM and lysis of the HaCaT cells was carried out with 1 mL of 1% (*v/v*) Tween-20 (SigmaAldrich, Darmstadt, Germany) at 37 °C for 30 min. Subsequently, dilutions of the *S. lugdunensis* suspension in each well were made and seeded on Trypton Soy Agar plates. After 18 h incubation at 37 °C, the number of viable cells was determined and compared to the control (untreated HaCaT cells).

For invasion assay, parallel experiments were carried out as described above. The exception was that in this part the plates were incubated with *S. lugdunensis* for 2 h in order to allow enough time for the bacteria to invade HaCaT cells, after which the cells were treated with gentamicin (300 µg/mL) for 1 h. Adherent cells were washed with FBS free DMEM three times, followed by incubation of the infected cells with 1% (*v/v*) Tween-20. Samples were diluted and plated on Trypton Soy Agar plates. After 18 h incubation at 37 °C, the number of viable cells was determined and compared to the control (untreated HaCaT cells).

### 2.9. Statistical Analysis

Results were presented as the mean ± standard deviation (SD) and significant differences (*p* < 0.05) were determined by One-way ANOVA with post-hoc Tukey HSD, using Xlstat 2018 program.

## 3. Results and Discussion

In the current study, extracts of two *Seseli* species were tested for their bioactive contents in terms of polyphenolic constituents. In order to assess the phenolic content, both spectrophotometric standard methods and HPLC techniques were used. The *Seseli* extracts in present study yielded TPC ranging from 19.09 to 24.33 mg GAE/g and TFC ranging from 0.45 to 10.09 mg RE/g (Table 1). The water extracts were found to yield much lower TFC than the methanolic extracts. In the phosphomolybdenum assay, the highest antioxidant capacity was obtained for the methanolic extract of *S. transcaucasicum*, followed by the methanolic extract of *S. gummiferum* (1.18 ± 0.12 and 0.90 ± 0.09 mmol TE/g, respectively). The total antioxidant capacity of the water extracts of *S. gummiferum* and *S. transcaucasicum* were 0.78 and 0.66 mmol TE/g, respectively (Table 1). The higher total antioxidant capacity of the methanolic extracts could be related to the higher TFC and slightly higher TPC than the water extracts.

Indeed, the choice of solvents is one the most important steps in phytochemical studies and at this point, the chemical composition and biological activities of the tested *Seseli* species were influenced by the solvent used. These findings could be a starting point for further studies on the tested *Seseli* species.

The TPC and TFC present in other *Seseli* species have also been determined. Notably, the TPC and TFC in the methanolic extracts of *S. pallasii Besser*, *S. libanotis* (L.) Koch ssp. *libanotis* and *S. libanotis* (L.) Koch ssp. *intermedium* (Rupr.) P. W. Ball ranged from 84.04–87.53 mg GAE/g and 4.76–19.38 mg quercetin/g, respectively, in the study of Matejić and colleagues [27]. In another study, the contents of total phenolics and flavonoids of various extracts of *S. rigidum* Waldst. Et Kit. (aqueous, acetone, ethyl acetate, methanol and petroleum ether) were evaluated, whereby the acetone extract was found to have the highest TPC and TFC (102.13 mg GAE/g and 291.58 mg RE/g, respectively) [28]. The different levels of bioactive contents could be explained by geographical and climatic factors (altitude, rainfall, etc.) and experimental differences (concentration, expressed unit, etc.) [29,30]. In addition, most scientists have recently given up the use of the Folin-Ciocalteu assay because not only phenolics but also other phytochemicals could play a reducing role in the assay [31]. Thus, the content of total phenolics has to be confirmed by at least one chromatographic technique and for this reason, phenolics were identified and quantified using HPLC, in the present study.

Based on HPLC quantification, a total of 18 constituents were found to be present in all the studied extracts except *S. gummiferum* water extract, in which only 17 compounds were identified. While naringenin was present in all the other extracts, it was absent in the *S. gummiferum* water extract. Chlorogenic acid was detected as the major constituent in methanolic and water extracts of *S. gummiferum*, as well as water extract of *S. transcaucasicum* (210.96, 198.53 and 107.48 µg/g, respectively). On the other hand, narcissin was found to be the most abundant constituent in the methanolic extract of *S. transcaucasicum* (261.40 µg/g), although chlorogenic acid was also present in high amount (243.12 µg/g) (Table 2). The UHPLC–DAD chromatograms at 280 nm, with some labelled peaks of polyphenols, are presented in Figure S1 (Supplementary Material). Interestingly, the methanolic extracts of the two *Seseli* species were observed to contain higher amounts of chlorogenic acid and narcissin compared to the water extracts, thus implying that methanol was a better solvent for extraction of these compounds than water. Accumulating evidence has demonstrated chlorogenic acid to display many biological properties, including the ability to modulate glucose and lipid metabolism in vivo in both healthy and genetically metabolic disordered conditions [32,33,34]. Other researchers have also previously shown interests in elucidating the chemical profile of different extracts and oils from *Seseli* species [35,36,37,38,39].

The antioxidant properties of plant-derived products have become an indispensable tool in determining their efficacy as treatments [4]. An imbalance between the synthesis and accumulation of reactive oxygen species (ROS) in cells and tissues, as well as the ability of a biological system to de-toxify these reactive products, causes oxidative stress [40]. Chronic illnesses such as diabetes, cardiovascular disease, neurodegenerative disease, and cancer all include oxidative stress as a pathogenesis factor [41]. Plants have been discovered to have an intrinsic ability to biosynthesize a wide spectrum of non-enzymatic antioxidants that can reduce ROS-induced oxidative damage [42]. As a result, plant-derived antioxidant molecules and their antioxidant defence mechanisms can help in the prevention of various illnesses. Antioxidant capacity is widely measured using in vitro antioxidant assays, which allow the examination of a wide range of potential antioxidant processes exhibited by phytochemicals such as polyphenols [43].

In the present investigation, all extracts were found to possess radical scavenging potential (DPPH: 5.51–11.45 mg TE/g; ABTS: 43.46–51.91 mg TE/g) (Table 3). However, extracts of *S. gummiferum* showed relatively higher scavenging potential compared to *S. transcaucasicum*. Moreover, reducing activity was demonstrated in both CUPRAC and FRAP assays (41.67–53.20 mg TE/g and 31.26–34.14 mg TE/g, respectively) (Table 3). In the CUPRAC assay particularly, the methanolic extracts displayed higher reducing potential compared to the water extracts. Metal chelating activity was also determined with activity ranging from 14.38 to 38.57 mg EDTAE/g (Table 3). Interestingly, the water extracts showed higher chelating potential than the methanolic extracts.

Several *Seseli* species have been previously validated for their antioxidant capacity. In the study of Önder, et al. [44], the radical scavenging activity and anti-lipid peroxidation of the ethyl acetate and methanolic extracts obtained from several *Seseli* species growing in Turkey namely, *S. andronakii*, *S. campestre*, *S. corymbosum*, *S. gummiferum* subsp. *gummiferum*, *S. hartvigii*, *S. libanotis*, *S. petraeum*, *S. arenarium*, *S. resinosum*, and *S. tortuosum* were evaluated (in DPPH assay: IC_50_ = 0.086–4.27 mg/mL; in lipid peroxidation assay: % inhibition of 43–95% at 5 mg/mL). Extracts of different polarity obtained from various plant parts (root, leaf, flower and fruit) of *S. rigidum* were also studied by different antioxidant assays such as DPPH and ABTS radical scavenging activity, total reducing power method as well as via total contents of flavonoids and polyphenols [37].

Indeed, it is important to point out that the performed assays are not independent of solvents used. For example, Foti [45] reviewed the solubility and kinetic properties of DPPH and DPPH was found to be poorly soluble in apolar solvents and generally polar solvents, especially methanol or ethanol, which are the most preferred for preparing DPPH. Moreover, ABTS is not a stable radical and it has been used to evaluate both lipophilic and hydrophobic antioxidant components in plant extracts. As another insight, FRAP and CUPRAC assays are pH dependent. Taken together, the antioxidant results could not be explained by just one factor as it could be influenced by several factors [46].

However, the phenolic compounds might be the major contributors to the obtained antioxidant results. This can be attributed to the presence of some phenolics, particularly chlorogenic acid, narcissin, epigallocatechin and aesculin. These compounds have been reported by several authors as significant antioxidants. For example, chlorogenic acid acts as a hydrogen donator to scavenge free radicals and inhibit oxidation reactions [47,48]. Noaves et al. [49] reported that narcissin exhibited remarkable ABTS radical scavenging ability with the value of 0.88 mol TE/mol of flavonol. In another study conducted by He et al. [50], different catechin derivatives were investigated for antioxidant properties and epigallocatechin displayed stronger DPPH radical scavenging ability than gallocatechin, epicatechin and catechin. In this sense, the number and position of the hydroxyl groups in the phenolic ring are directly related to their antioxidant capacities [51].

The use of enzyme inhibitors in drug discovery has become a common practice in pharmacology. Enzymes play a role in a wide variety of human diseases and a number of specific enzyme inhibitors have been developed to inhibit their activity and therefore act as therapeutic agents [52].

The enzyme cholinesterase (ChE) is an important therapeutic target for Alzheimer’s disease (AD). The deterioration of cholinergic neurons in the brain and the loss of neurotransmission are the main causes of the decline in cognitive function in AD patients. Thus, one of the potential therapeutic strategies is to boost the cholinergic levels in the brain by inhibiting the biological activity of ChE [53]. In the current investigation, all the studied extracts inhibited both cholinesterases and the same order of inhibitory potency of the extracts was observed against the enzymes, that is *S. transcaucasicum*-MeOH > *S. gummiferum*-MeOH > *S. gummiferum*-Water > *S. transcaucasicum*-Water (AChE: 0.15–4.53 mg GALAE/g and BChE: 1.66–9.71 mg GALAE/g) (Table 4). In particular, the methanolic extracts possessed higher anti-cholinesterase activities compared to the water extracts. When chemical components and cholinesterase inhibitory properties were analysed, the presence of some phenolic components was found to support the observed activity. For example, chlorogenic and caffeic acids were reported as significant acetylcholinesterase inhibitors in an earlier study performed by Oboh et al. [54]. In addition, rosmarinic acid [55], gallic acid [56] and rutin [57] showed remarkable cholinesterase inhibitory effects.

Inhibition of tyrosinase, a copper-containing enzyme that catalyses the essential stages of melanogenesis, is one of the most popular ways to control skin pigmentation [58]. Current research has focused on both synthetic and natural sources for reducing melanin formation. With respect to natural substances, many plant species have been found to be major sources of skin lightening agents [59,60]. In the present study, only the methanolic extracts were found to possess anti-tyrosinase activity, while the water extracts were inactive against tyrosinase. For instance, the tyrosinase inhibitory activity of the methanolic extracts of *S. transcaucasicum* and *S. gummiferum* was recorded as 109.37 ± 0.38 mg KAE/g and 107.15 ± 1.38 mg KAE/g, respectively (Table 4). The higher tyrosinase inhibitory properties can be associated with higher total phenolics in the methanolic extracts. In particular, the presence of some phenolics, including chlorogenic, caffeic and rosmarinic acids might have contributed to the observed tyrosinase inhibitory abilities. Consistent with our presented results, these compounds have been reported as significant tyrosinase inhibitors in previous studies [61,62,63]. Taken together, the tested methanolic extracts could be considered as a source of natural tyrosinase inhibitors for cosmeceutical applications.

The inhibition of α-glucosidase and α-amylase, enzymes involved in carbohydrate digestion, can considerably minimize the postprandial glucose level and hence can be a useful technique in the control of blood glucose levels in type 2 diabetic individuals [64]. Therefore, several studies have been performed to establish the antidiabetic potential of extracts or isolated components from different plant species [65,66]. In this study, all extracts were found to inhibit amylase exclusively, except the water extract of *S. gummiferum* which also inhibited glucosidase(0.13 ± 0.01 mmol ACAE/g). Moreover, the methanolic extracts showed higher anti-amylase activity compared to the water extracts. Besides, both methanolic extracts and both water extracts showed the same inhibitory activity against the amylase (0.78 mmol ACAE/g and 0.12 mmol ACAE/g, respectively) (Table 4). Recently, several papers have indicated that phenolics are of great interest as carbohydrate-hydrolyzing enzyme inhibitors. For example, chlorogenic acid exhibited a good amylase inhibition action with an IC_50_ value of 0.498 mg/mL [67]. Similar findings were also reported for rosmarinic, caffeic and gallic acids [67,68,69]. Interestingly, many flavonoids have been found to possess anti-carbohydrate hydrolysing effects. Tadera, et al. [70], for instance, tested several flavonoid compounds for their inhibitory activity against α-amylase. They showed that luteolin, myricetin, and quercetin were potent inhibitors against porcine pancreatic α-amylase and that the potency of the inhibition correlated with the number of hydroxyl groups on the B ring of the flavonoid scaffold.

Overall, the methanolic extracts were revealed to be better inhibitors of the studied enzymes compared to the water extracts, which may be due to the presence of certain active compounds that were extracted in methanol and not water. Our findings could open new horizons for the development of novel pharmaceuticals using natural enzyme inhibitors and the tested *Seseli* species could be considered as raw materials in this sense.

As revealed by the cytotoxic studies of the extracts on HaCaT cells, the water extracts were found to be more cytotoxic (IC_50_: 267.8 ± 4.14 µg/mL and 321.41 ± 1.25 µg/mL for *S. gummiferum* and *S. transcaucasicum*, respectively) than the methanolic extracts (IC_50_: >500 µg/mL) (Table 5).

Interestingly, previous scientific reports also investigated the cytotoxic effects of *Seseli* species on different cell lines, including cancer cell lines. For example, the extracts obtained from the root of *S. petraeum* showed a significant inhibitory effect on cell proliferation. The hexane extract of the root exhibited potent inhibition on A549 cancer cell growth at the 24th h (IC_50_: 3.432 mg/mL). The results also showed that the hexane extract displayed the cytotoxic effect through an arrest at the G0/G1 phase of the cell cycle and induced apoptosis as well as DNA damage in A549 cells [8]. Besides, a recent study showed that chlorogenic acid, a main compound identified in the *Seseli* species in the current study, did not reduce viability of human promyelocytic leukemia cells (HL-60) and human acute T-cell leukemia cells (Jurkat). Furthermore, chlorogenic acid at a concentration of 100 μM induced global DNA hypomethylation, a cell-specific effect in Jurkat cells [71]. Another study showed that chlorogenic acid exhibited cytotoxic activity via hydrogen peroxide-mediated oxidation mechanism in human oral squamous cell carcinoma (HSC-2) and salivary gland tumor (HSG) cell lines [72].

Following the cytotoxic study, the wound healing potential of the extracts was evaluated. While the methanolic extracts of both *Seseli* species were noted to have a wound healing effect (Relative cell migration: 31.23 ± 2.24% and 28.21 ± 2.64% for *S. gummiferum* and *S. transcaucasicum*, respectively), the water extracts did not have any effect on cell migration into the wounded area (Table 6). *S. gummiferum* methanolic extract showed a relatively higher wound healing effect than that of *S. transcaucasicum*. However, while wound healing effect could be noted for the methanolic extracts of these two species, it was not so prominent. Chlorogenic acid was represented by higher amounts in methanolic extracts of the *Seseli* species. This is interesting because the wound healing potential was noted for the extracts that contained more chlorogenic acid and narcissin. Previous findings suggested that chlorogenic acid enhanced the capillary-like tube formation of endothelial cells in an in vitro angiogenesis assay and accelerated the fibroblastic and remodelling phases [73]. It is well known that notable wound healing activity of chlorogenic acid is brought about by the enhancement of collagen synthesis via upregulation of Tumor Necrosis Factor-α and transforming Growth Factor-β1 in different phases of wound healing, and also because of its antioxidant potential [74]. Therefore, the wound healing potential of the investigated species might be partly attributed to the presence of chlorogenic acid.

The discovery of new antimicrobial compounds is important in controlling bacterial infections caused by some pathogens that are rapidly becoming resistant to many existing antibiotics. In this context, folk medicine as an alternative form of health care, together with the development of microbial resistance to available antibiotics have led scientists to study the antimicrobial activity of medicinal plants [75].

In the present study, all extracts showed antibacterial activity against *S. lugdunensis* (MIC: 0.025–1 mg/mL; MBC: 0.5–2 mg/mL), although they were not as potent as the antibiotic streptomycin used as positive control in this study (MIC: 0.003 mg/mL; MBC: 0.006 mg/mL) (Table 7). The methanolic extract of *S. gummiferum* and the water extract of *S. transcaucasicum* demonstrated the highest and lowest antibacterial potential, respectively. It was interesting to note that the phytochemical composition of the investigated species could be linked to their antimicrobial activity. For instance, recent findings [76] have pointed out the antimicrobial activity of chlorogenic acid against the *Salmonella* species. The mechanism of antibacterial action involved damage of intracellular and outer membranes as well as disruption of cell metabolism, which resulted into death of the bacteria. Therefore, the obtained antimicrobial effect could be attributed to predominant constituents, including chlorogenic acid.

**Figure 1:**
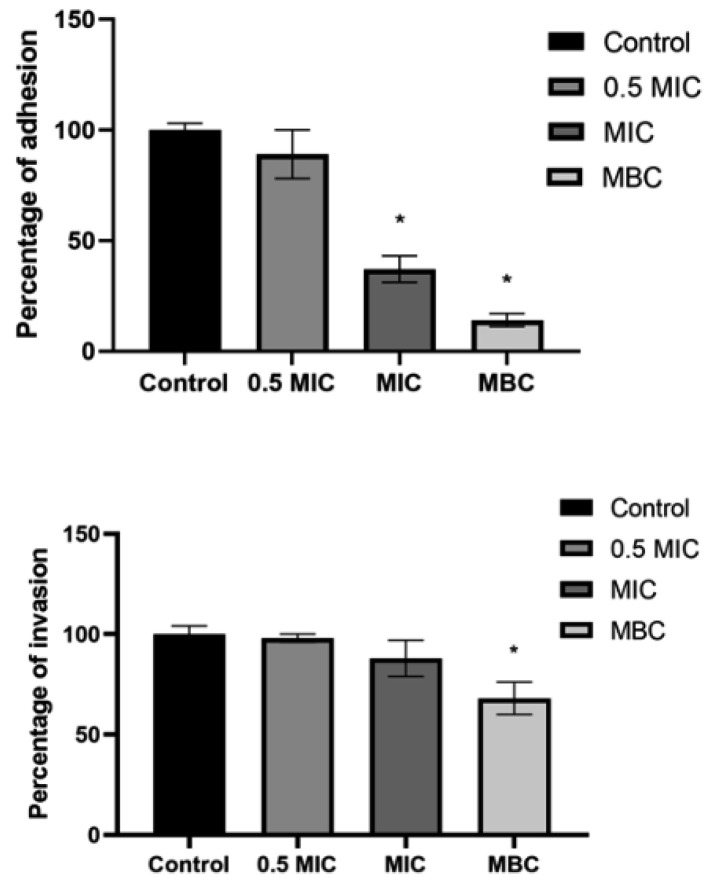
Adhesion and invasion (%) of *S. lugdunensis* to HaCaT cells after treatment with *S. gummiferum*-MeOH (0.5 MIC-MBC) compared to the untreated control (100%). Students *t*-test was used (GraphPad Prism 9.0.0.); the asterisks represent statistical significance *, *p* ≤ 0.05.

The adhesion and invasion of *S. lugdunensis* to HaCaT cells after treatment with methanolic extract of *S. gummiferum*, which showed the most potent antibacterial activity among all the extracts, were also monitored. For instance, at MIC, the % of adhesion and invasion were <40% and <90%, respectively, while at MBC, the % of adhesion and invasion were <20% and <70%, respectively, compared to the untreated control, which showed 100% adhesion and invasion of *S. lugdunensis* (Figure 1).

The members of the genus *Seseli* have been previously considered for antimicrobial studies. For instance, the essential oil of *S. annuum* showed antifungal activity against fifteen fungi (MIC values: 12.5–50 μL/mL) [9]. Similarly, the essential oil from the aerial parts of *S. globiferum* Vis. displayed significant activities both against bacteria (*Pseudomonas aeruginosa*, *Micrococcus flavus*, *Listeria monocytogenes* and *Escherichia coli*) and all investigated fungal species in a study conducted by Janaćković et al. [36]. Moreover, in the study of Stankov-Jovanović et al. [37], the inhibitory and microbicidal concentration ranges of the tested extracts of *S. rigidum* against the bacteria *Escherichia coli*, *Pseudomonas aeruginosa*, *Staphylococcus aureus*, *Bacillus cereus*, and fungi *Candida albicans* and *Aspergillus niger* were 0.01–1.50 mg/mL and 0.02–3.00 mg/mL, respectively. It was also reported by Tosun, et al. [77] that the n-hexane extracts of *S. resinosum* and *S. hartvigii* and the essential oil of *S. gummiferum* subsp. *gummiferum* displayed antimicrobial activity against two strains of *Staphylococcus aureus*. Furthermore, Ozturk and Ercisli [78] investigated the antibacterial properties of the methanolic extract of *S. libanotis* against several strains and remarkable antibacterial abilities were reported (>14 mm inhibition zone).

## 4. Conclusions

This study is the first comparative report on the biological and chemical properties of *S. gummiferum* and *S. transcaucasicum* water and methanolic extracts obtained from the aerial parts of the plants. The studied *Seseli* extracts were found to possess multitargeted pharmacological potentials, as evidenced in various assays performed. For instance, they displayed antioxidant effects and acted as inhibitors of enzymes having a key role in the pathogenesis of diseases such as diabetes, Alzheimer’s disease and skin hyperpigmentation. Following their cytotoxic studies on HaCaT cells, the extracts were also tested for wound healing potential, whereby only the methanolic extracts showed very little effect. Moreover, they displayed inhibitory and bactericidal action on the bacterium *S. lugdunensis*. The methanolic extract of *S. gummiferum* which showed the highest antibacterial potency, was also found to inhibit adhesion and invasion of *S. lugdunensis* to HaCaT cells. The extracts were also revealed to be rich sources of polyphenolic compounds. The methanolic extracts in particular were noted to contain chlorogenic acid and narcissin in greater quantity compared to the water extracts. However, choosing an appropriate solvent for the extraction of plant materials is of high importance for the yield of certain compounds. Since the biological activities of the extracts are linked with the quantity of compounds present and their ratios in the extracts, it might be concluded that biological activity of the extracts is directly dependent on the plant species and extraction solvents used. Overall, this study demonstrated the extracts to exhibit important biological potentials and could be exploited as health promoting agents. Nevertheless, in vivo studies would be helpful to further investigate the toxicity and safe use of these extracts to benefit from their healing potentials.

## Figures and Tables

**Table 1:** Total bioactive contents and total antioxidant capacity (by phosphomolybdenum assay) of the tested extracts.

Extracts	TPC (mg GAE/g)	TFC (mg RE/g)	PDA (mmol TE/g)
*S. gummiferum*-MeOH	24.33 ± 0.84 ^a^	7.33 ± 0.50 ^b^	0.90 ± 0.09 ^b^
*S. gummiferum*-Water	21.18 ± 0.14 ^b^	0.48 ± 0.04 ^c^	0.78 ± 0.09 ^b^
*S. transcaucasicum*-MeOH	23.72 ± 0.69 ^a^	10.09 ± 0.20 ^a^	1.18 ± 0.12 ^a^
*S. transcaucasicum*-Water	19.09 ± 0.09 ^c^	0.45 ± 0.04 ^c^	0.66 ± 0.11 ^b^

Values are reported as mean ± SD. MeOH: Methanol; TPC: total phenolic content; TFC: total flavonoid content; GAE: Gallic acid equivalent; RE: rutin equivalent; TE: trolox equivalent. PDA: Phosphomolybdenum assay. Different letters indicate significant differences among the tested extracts (*p* < 0.05).

**Table 2:** Polyphenolic constituents detected in extracts (µg/g extract) by means of UHPLC(-)HESI-QqQ-MS/MS.

Polyphenolic Constituents (µg/g)	*S. gummiferum*-MeOH	*S. gummiferum*-Water	*S. transcaucasicum*-MeOH	*S. transcaucasicum*-Water
Gallic acid	37.81	22.27	31.94	8.70
Protocatechuic acid	61.33	66.32	73.02	66.25
Syringic acid	51.53	53.35	57.80	54.19
Aesculin	56.41	59.77	70.60	58.97
Epigallocatechin	51.28	60.91	75.05	40.98
Chlorogenic acid	210.96	198.53	243.12	107.48
Aesculetin	8.05	6.89	11.15	4.77
Caffeic acid	11.86	11.65	15.37	5.38
Isoorientin	3.80	3.10	5.79	4.38
Rutin	15.22	14.42	54.52	26.00
p-Coumaric acid	18.69	27.16	28.93	24.67
Isoquercetin	8.67	6.87	21.56	8.73
Narcissin	202.13	57.61	261.40	61.29
Quercitrin	2.20	2.49	4.59	2.26
Rosmarinic acid	8.20	4.91	2.98	2.73
Apigenin	2.27	2.35	3.33	2.45
Naringenin	2.05	NF	3.33	2.80
Hispidulin	5.18	1.94	9.49	9.70
NF—not found				

**Table 3:** Antioxidant properties of the tested extracts.

Extracts	DPPH (mg TE/g)	ABTS (mg TE/g)	CUPRAC (mg TE/g)	FRAP (mg TE/g)	MCA (mg EDTAE/g)
*S. gummiferum*-MeOH	10.24 ± 0.67 ^a,b^	51.91 ± 0.81 ^a^	53.20 ± 2.38 ^a^	33.32 ± 0.57 ^a,b^	15.93 ± 0.45 ^c^
*S. gummiferum*-Water	11.45 ± 0.77 ^a^	46.78 ± 1.03 ^b^	44.28 ± 1.18 ^b,c^	34.14 ± 0.14 ^a^	22.76 ± 0.36 ^b^
*S. transcaucasicum*-MeOH	5.51 ± 0.94 ^c^	43.46 ± 0.97 ^c^	48.07 ± 1.58 ^b^	32.44 ± 0.99 ^b,c^	14.38 ± 0.24 ^d^
*S. transcaucasicum*-Water	8.53 ± 0.38 ^b^	45.70 ± 0.88 ^b,c^	41.67 ± 0.33 ^c^	31.26 ± 0.52 ^c^	38.57 ± 0.93 ^a^

Values are reported as mean ± SD. ABTS: 2,2′-azino-bis(3-ethylbenzothiazoline-6-sulphonic acid; DPPH: 1,1-diphenyl-2-picrylhydrazyl; CUPRAC: Cupric reducing antioxidant capacity; FRAP: Ferric reducing antioxidant power; MCA: Metal chelating activity. MeOH: Methanol; TE: Trolox equivalent; EDTAE: EDTA equivalents. Different letters indicate significant differences among the tested extracts (*p* < 0.05).

**Table 4:** Enzyme inhibitory effects of the tested extracts.

Extracts	AChE (mg GALAE/g)	BChE (mg GALAE/g)	Tyrosinase (mg KAE/g)	Amylase (mmol ACAE/g)	Glucosidase (mmol ACAE/g)
*S. gummiferum*-MeOH	3.48 ± 0.09 ^b^	8.90 ± 0.35 ^a^	107.15 ± 1.38 ^b^	0.78 ± 0.01 ^a^	na
*S. gummiferum*-Water	0.35 ± 0.08 ^c^	6.52 ± 0.97 ^b^	na	0.12 ± 0.02 ^b^	0.13 ± 0.01
*S. transcaucasicum*-MeOH	4.53 ± 0.19 ^a^	9.71 ± 0.47 ^a^	109.37 ± 0.38 ^a^	0.78 ± 0.01 ^a^	na
*S. transcaucasicum*-Water	0.15 ± 0.05 ^d^	1.66 ± 0.17 ^c^	na	0.12 ± 0.01 ^b^	na

Values are reported as mean ± SD. AChE: acetylcholinesterase; BChE: butyrylcholinesterase; MeOH: Methanol; GALAE: Galantamine equivalent; KAE: Kojic acid equivalent; ACAE: Acarbose equivalent; Na: not active. Different letters indicate significant differences among the tested extracts (*p* < 0.05).

**Table 5:** Cytotoxicity of extracts towards HaCaT cells.

Extracts	IC_50_ (µg/mL)
*S. gummiferum*-MeOH	>500
*S. gummiferum*-Water	267.8 ± 4.14
*S. transcaucasicum*-MeOH	>500
*S. transcaucasicum*-Water	321.41 ± 1.25

**Table 6:** Relative number of single cells that migrated into wound region (%).

Extract	Wound Healing (%)
*S. gummiferum*-MeOH	31.23 ± 2.24
*S. gummiferum*-Water	NE
*S. transcaucasicum*-MeOH	28.21 ± 2.64
*S. transcaucasicum*-Water	NE
Control	26.37 ± 3.56

NE—no effect on migration (less than untreated control).

**Table 7:** Antibacterial activity of extracts against *S. lugdunensis*, results are in mg/mL.

Extract	*Staphylococcus lugdunensis*	
	MIC	MBC
*S. gummiferum*-MeOH	0.025	0.5
*S. gummiferum*-Water	0.5	1
*S. transcaucasicum*-MeOH	0.5	1
*S. transcaucasicum*-Water	1	2
Streptomycin	0.003	0.006

MIC: minimum inhibitory concentration; MBC: minimum bactericidal concentration.

## Data Availability

All data is contained within the article and Supplementary Materials.

## Supplementary Materials

The following are available online at https://www.mdpi.com/article/10.3390/antiox10101510/s1, Figure S1: The UHPLC–DAD chromatograms at 280 nm, with some labeled peaks of quantified polyphenols; (1) gallic acid; (2) chlorogenic acid; (3) isoorientin; (4) rutin; (5) *p*-coumaric acid; (6) isoquercetin; (7) narcissin; (8) rosmarinic acid; (A) *S. gummiferum* MeOH extract; (B) *S. gummiferum* water extract; (C) *S. lugdunensis* MeOH extract; (D) *S. lugdunensis* water extract.
