# Correction: Zengin et al. Comprehensive Biological and Chemical Evaluation of Two *Seseli* Species (*S. gummiferum* and *S. transcaucasicum*). *Antioxidants* 2021, *10*, 1510

**DOI:** 10.3390/antiox15010047

**Published:** 2025-12-30

**Authors:** Gokhan Zengin, Dejan Stojković, Mohamad Fawzi Mahomoodally, Bibi Sharmeen Jugreet, Mehmet Yavuz Paksoy, Marija Ivanov, Uroš Gašić, Monica Gallo, Domenico Montesano

**Affiliations:** 1 Department of Biology, Science Faculty, Selcuk University, 42130 Konya, Turkey; 2 Department of Plant Physiology, Institute for Biological Research “Siniša Stanković”—National Institute of Republic of Serbia, University of Belgrade, Bulevar Despota Stefana 142, 11000 Belgrade, Serbia; marija.smiljkovic@ibiss.bg.ac.rs (M.I.); uros.gasic@ibiss.bg.ac.rs (U.G.); 3 Department of Health Sciences, Faculty of Medicine and Health Sciences, University of Mauritius, Réduit 80837, Mauritius; f.mahomoodally@uom.ac.mu (M.F.M.); sharmeenjugs@gmail.com (B.S.J.); 4 Department of Medical Services and Techniques, Medical Documentation and Secretaryship Programme, Tunceli Vocational School, Munzur University, 62000 Tunceli, Turkey; mypaksoy@gmail.com; 5 Department of Molecular Medicine and Medical Biotechnology, University of Naples Federico II, Via Pansini 5, 80131 Naples, Italy; mongallo@unina.it; 6 Department of Pharmacy, University of Naples Federico II, Via D. Montesano 49, 80131 Naples, Italy

In the original publication [1], there was a mistake in Figure 1 as published. The figure, as published, did not include the appropriate statistical information. We have revised the figure accordingly, and the corrected version of “Figure 1” is presented below. The authors state that the scientific conclusions are unaffected. This correction was approved by the Academic Editor. The original publication has also been updated.

## Figures and Tables

**Figure 1:**
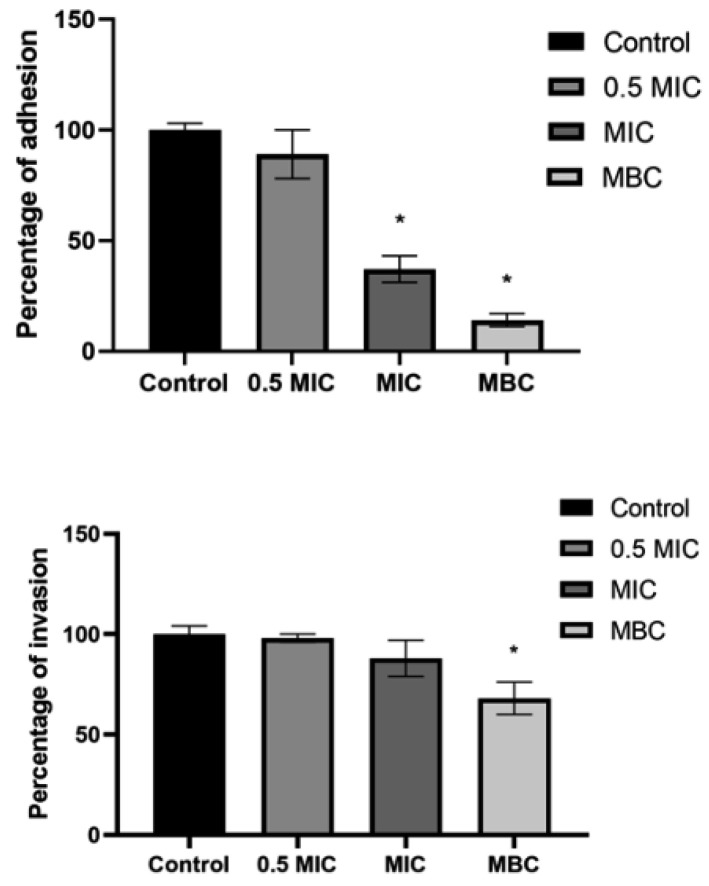
Adhesion and invasion (%) of *S. lugdunensis* to HaCaT cells after treatment with *S. gummiferum*-MeOH (0.5 MIC-MBC) compared to the untreated control (100%). Students *t*-test was used (GraphPad Prism 9.0.0.); the asterisks represent statistical significance *, *p* ≤ 0.05.
