# Supplementary material for: Comprehensive Biological and Chemical Evaluation of Two Seseli Species (S. gummiferum and S. transcaucasicum)
Source: Antioxidants (Basel). 2021 Sep 24;10(10):1510. doi: 10.3390/antiox10101510 (PMC8532678; doi:10.3390/antiox10101510)
Supplement: Supplementary file 1 [file antioxidants-10-01510-s001.zip › antioxidants-1373740-SI.pdf]

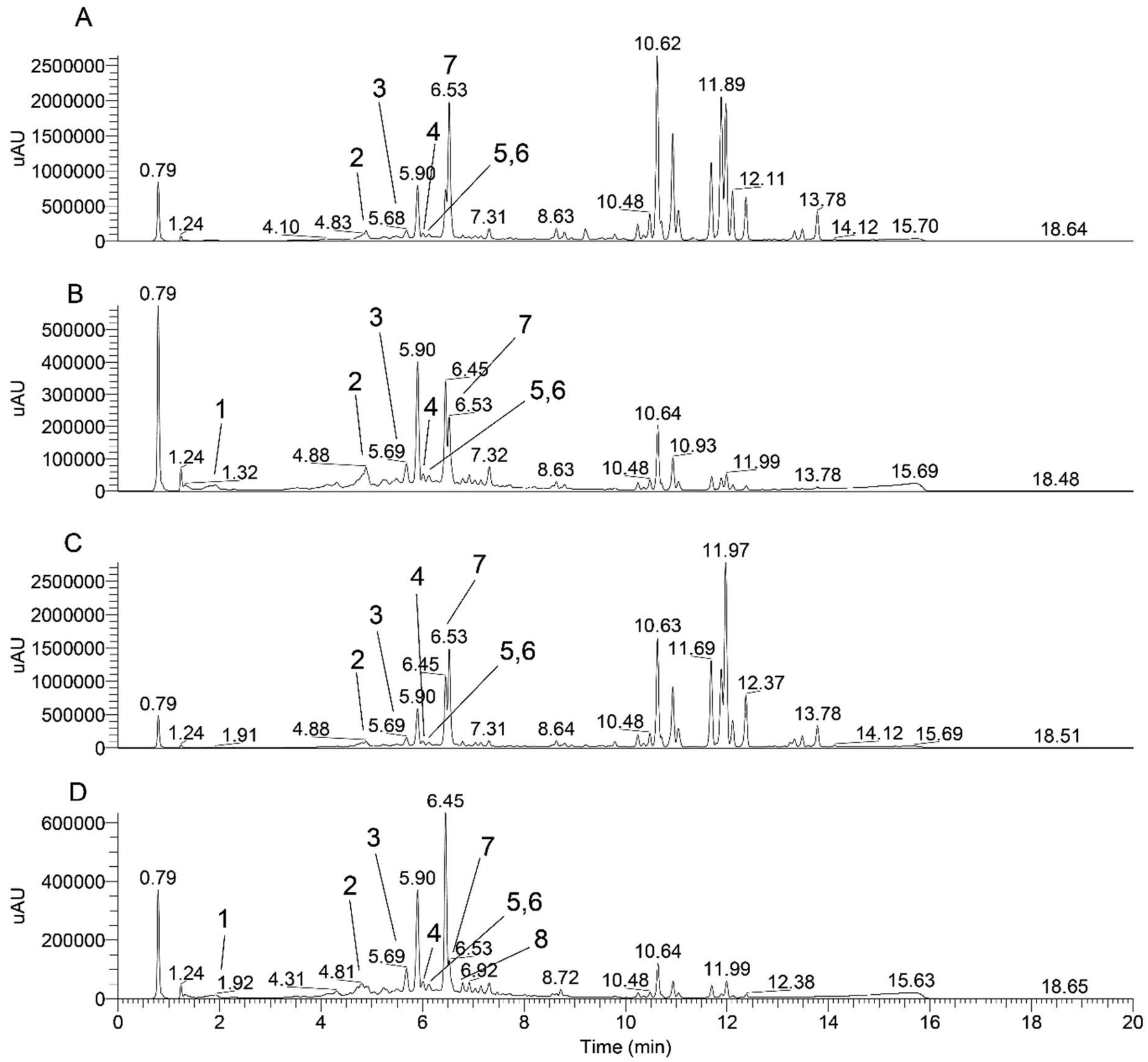

Figure S1. The UHPLC–DAD chromatograms at 280 nm, with some labeled peaks of quantified polyphenols; 1) gallic acid; 2) chlorogenic acid; 3) isoorientin; 4) rutin; 5) *p*-coumaric acid; 6) isoquercetin; 7) narcissin; 8) rosmarinic acid; A) *S. gummiferum* MeOH extract; B) *S. gummiferum* water extract; C) *S. lugdunensis* MeOH extract; D) *S. lugdunensis* water extract.
